# Lessons learned from a peri-urban needle exchange

**DOI:** 10.1186/1477-7517-7-8

**Published:** 2010-04-29

**Authors:** Andrea K Knittel, Patricia A Wren, Lemont Gore

**Affiliations:** 1 Department of Health Behavior and Health Education, School of Public Health, University of Michigan, Ann Arbor, MI, USA; 2 Wellness, Health Promotion, and Injury Prevention Program, School of Health Sciences, Oakland University, Rochester, MI, USA; 3 HIV/AIDS Resource Center, Ypsilanti, MI, USA

## Abstract

**Background:**

Injection drug users continue to be at high risk of HIV and HCV. Research has shown that needle exchange programs (NEP) decrease injection frequency, reduce syringe reuse, and reduce needle sharing, though some results have been mixed.

**Methods:**

This evaluation of a small, peri-urban, legal NEP near Ypsilanti, Michigan describes the operation of the NEP and its clients. It uses interviews conducted with NEP participants between 2003 and 2006, describing the population served by the program, and draws on limited comparisons between matched baseline and follow-up measures as well as aggregate baseline and follow-up comparisons.

**Results:**

The HIV/AIDS Resource Center (HARC) Harm Reduction NEP serves a diverse population from a wide geographical area. NEP participants at follow-up reused their syringes significantly fewer times before getting new ones, were significantly less likely to report giving another IDU a previously used syringe, and were more likely to clean their skin with alcohol either before or after injecting than the baseline comparison group.

**Conclusions:**

The limited data presented here suggest that a NEP can be an effective method of harm reduction even in low-volume, non-urban settings and are an important venue for intervention in peri-urban areas.

## Background

As of 2001 in the United States, approximately one-third of AIDS cases and one-half of new hepatitis C cases were associated with injection drug use (IDU) [1]. In 2006, injection drug use was a risk factor in approximately 16% of new HIV cases. Although new infections among IDUs declined in the past decade, IDUs remain a high risk population. IDUs and their sex partners represent approximately one-third of persons infected in the HIV epidemic and continue to be at risk for transmitting both HIV and HCV, necessitating continued prevention efforts [2]. These data highlight the significant problem of HIV risk and infection among IDUs in the United States.

In 1986, the first NEP in this country began in New Haven, CT [3]. Since then, and despite a ban on federal funding for NEPs that ended in late 2009, the number of NEPs and evidence of their effectiveness has grown significantly [4]. NEPs have been associated with substantially decreased odds of HIV risk behavior [5]. Research shows NEPs decrease injection frequency, reduce syringe reuse, and reduce needle sharing, though some results have been mixed [6-10]. In addition to reduction of injection risk, NEPs may be a valuable venue for sexual risk reduction interventions.

Although opponents of NEPs cite drug treatment as an alternative to harm reduction, there is some evidence that former needle exchange-using IDUs were more likely than never-exchangers to remain in drug treatment [7]. Evidence is mixed, however, and additional research is necessary to determine how best to promote access to drug treatment for NEP participants [11].

The focus of current research and evaluation has been on large urban NEPs such as those in New York City, New York, Baltimore, Maryland, and New Haven, Connecticut. While these programs represent a large percentage of syringes exchanged, there remains a gap in the literature. We hypothesize that even smaller programs in peri-urban cities will reduce HIV risk behaviors and show many of the same benefits demonstrated in larger programs.

The HIV/AIDS Resource Center (HARC) in Ypsilanti, Michigan runs a legal NEP in several surrounding cities and townships. HARC operates under the principles of harm reduction, acknowledging that individuals will continue to use substances until they determine a need or develop a willingness to change. HARC provides sterile syringes, safer injection materials, condoms, HIV testing and counseling, as well as a substance abuse specialist to coordinate entry into treatment programs in order to enable individuals to access evidence-based behavioral intervention alternatives without judgment or moralization. The HARC program serves individuals from a four-country region comprised mainly of small cities and townships, as well as some clients from more rural areas. This evaluation was conducted to determine whether a small NEP, a proven harm reduction program, would demonstrate behavioral risk reduction effects in a peri-urban area.

## Methods

During the summer of 2006, the first author spent several days each week as a volunteer with the HARC NEP. The description of the operation of the program is based on her impressions and discussions with HARC staff.

Between 2003 and 2006, HARC NEP staff interviewed needle exchange participants at their first visit and again at 6-months using an intake and follow-up questionnaire designed by HARC. Interviews were conducted in a private room in the HARC outreach van to maintain privacy and confidentiality. A structured survey form was employed to gather information from NEP clients.

The forms were altered slightly over time to increase the accuracy of the interview responses and to more precisely reflect the NEP goals. In total there were five questionnaire versions. Responses to items on each version of the questionnaire were coded to combine identical questions. Identical questions that appeared on some versions with a numerical frequency scale (i.e., none, 1-3 times, 4-6 times, 7-9 times, 10+ times) and on other versions with a subjective response scale (i.e., never, rarely, sometimes, often, always) were also combined.

Variables representing frequency of using a previously used needle, giving used syringes to another IDU, exchanging needles for another IDU, sharing other injection materials, cleaning skin with alcohol, reusing the same syringe, bleaching needles before reuse, condom usage, and HIV testing were dichotomized into "never" (included "zero" and "never" responses) or "ever" (included responses indicating they had previously been tested, previously received results, or had consented to being tested at the time of the interview) to reflect the potential for measurement differences based on the scales used in different versions of the questionnaires. A dichotomized item to represent the number of sexual partners reported was also created: either one partner or any number greater than one (those NEP users with no sexual partners were excluded from the analysis of this variable).

HARC used the Transtheoretical Model to measure progress toward changing drug-using behaviors [12]. Individual respondent's stage was measured using a single question regardless of the questionnaire version employed. Some versions explicitly stated the five stages (Pre-contemplation, Contemplation, Preparation, Action, or Maintenance) and asked respondents to pick the stage that best described them. Other versions used a proxy measure and asked respondents to select from a list of statements the one that best described them in order to represent the client's stage (i.e., not at risk, need to decrease risk, ready to change, started doing things, or have been doing things). Responses to all forms of this question were combined into a single scale and coded as follows: 1 = Pre-contemplation or Not at risk, 2 = Contemplation or Need to decrease risk, 3 = Preparation or Ready to change, 4 = Action or Started doing things, and 5 = Maintenance or Have been doing things.

The initial data analysis plan consisted of paired comparisons between baseline and follow-up measurements of injection-related and sexual behavior-related HIV risk behaviors. Descriptive analyses revealed that only a small number of subjects completed both baseline and follow-up interviews which changed the analysis plan significantly. As a result, the focus of the analysis was shifted to a description of the program and the population it serves.

Aggregated baseline interviews were compared with aggregated follow-up interviews using the dichotomized variables to examine general trends over time. Although there was potentially a correlation between the observations of the 14 individuals with both baseline and follow-up interviews, and a generalized estimating equation model was considered, independence was assumed because the correlated observations were only a small percentage of the total number of observations. Logistic regression was used to calculate unadjusted odds ratios for the dichotomized variables using baseline measurement as the reference category, and simple linear regression was used to calculate coefficients, confidence intervals, and p-values for continuous and ordinal categorical variables. The logistic regression models initially included only the baseline/follow-up variable as a predictor. Race and sex, when added to the model, were not significant and not retained in further models. Odds ratios were calculated for the follow-up group compared to the baseline group. The small paired sample was analyzed using paired t-tests on non-dichotomized variables (except for whether the individual had ever had an HIV test, which was dichotomized into never or ever) to assess differences between the two groups and to generate hypotheses for further investigation. SPSS and R statistical packages were used for statistical analysis.

This study was approved by the Medical School Institutional Review Board (IRBMED - HUM00004528) and reapproved by the Health Sciences and Behavioral Sciences IRB at the University of Michigan after a change in university affiliation.

## Results

### Description of the NEP

The HARC NEP was started in December of 2000 and is run exclusively from the outreach van that parks three days a week in designated locations to provide easy access for injection drug users. The NEP provides a wide range of services, including HIV testing, safer sex materials, bleach kits, hygiene kits, risk reduction counseling and referrals to needed services including substance abuse treatment. The actual exchange of used syringes for clean ones occurs in a private part of the van not visible to other clients approaching the van to obtain condoms, other safer sex materials, or information from the volunteers and staff. This private space is also used for HIV counseling and testing, in-depth risk reduction counseling, and sometimes testing for hepatitis C provided by the health department.

Availability of the needle exchange program at outreach van sites has varied over time due to local politics and jurisdiction boundaries. Although several sites have consistently allowed legal needle exchange, many of the small townships have decided in recent years that needle exchange is unnecessary in their communities - often independent of any data on injection drug use in those same communities. Due to privacy and legal concerns, participants in the NEP are rarely willing to speak at community meetings about their need for needle exchange, making evidence-based policy-making difficult. The dearth of anecdotal and experiential information is further compounded by a lack of quantitative data for many of the same reasons. Also complicating the operation of the NEP is its coverage of a wide array of jurisdictions, including several cities and townships that each requires their own permissions and approvals.

In spite of these difficulties, many NEP clients are long-time users of the program, following it from site to site, and have developed relationships with the NEP staff. The ability of the NEP staff to maintain confidentiality as well as rapport with the clients is an asset to the program and allows for continuity even in the face of political vulnerability.

### Description of the NEP Participants

Table 1 presents the response rates for NEP participants. Eighty-eight individuals were surveyed at baseline and these included all unique ID numbers for respondents between 2003 and 2006. Of these, 74 (84%) completed only a baseline interview while 14 individuals (16%) completed both a baseline and follow-up interview. A total of 31 individuals completed follow-up interviews. Of these, 17 respondents (55%) completed a follow-up interview having utilized NEP services for a time without first completing a baseline interview.

**Table 1:** Response rates

	n	%
Overall baseline sample	88	(100)

Baseline only	74	(84)

		

Overall follow-up sample	31	(100)

Follow-up only	17	(55)

		

Baseline and follow-up	14	(16)

Table 2 gives a demographic description of the NEP participants. The participants were primarily male (78.6% and 52.9% male at baseline and follow-up respectively), though the proportion of female participants at follow-up was appreciably higher. The median age was approximately 50 years old, with an inter-quartile range of 43-56 years in the baseline only group, 44-52 in the follow-up only group, and 49-56 among those participants who completed both a baseline and follow-up interview. Slightly more than half of the population served by the NEP is Black or African American, though there is a high rate of unreported race/ethnicity measures, so this should only be considered an estimate.

**Table 2:** Demographic description of study sample

	Baseline Only	Follow-Up Only	Both
Total sample size	74	17	14

			

% male	78.3	52.9	78.6

			

Age in 2006	(years)	(years)	(years)

Mean (SD)	48 (12)	47 (9)	54 (8)

1^st ^Quartile	56	52	56

Median	51	48	52

3^rd ^Quartile	43	44	49

			

Race/Ethnicity	N (%)	N (%)	N (%)

Black/African American	40 (54.0)	2 (11.8)	8 (57.1)

White/Caucasian	32 (43.0)	0 (0)	5 (35.7)

Native American/Alaskan Native	2 (2.7)	0 (0)	0 (0)

Not recorded	0 (0)	15 (88.2)	1 (7.1)

			

% zip 48197/8	71.6	87.5	100

Participants in the NEP live primarily in zip codes 48197 and 48198, closest in proximity to the NEP sites. These two zip codes correspond with the city of Ypsilanti, with 22,362 residents, as well as the townships and villages further out from the city center [13]. Ypsilanti is located approximately 12 miles east of Ann Arbor (itself a city of approximately 114,000), and 35 miles west of Detroit. The other zip codes given by NEP participants show that HARC provides services to individuals coming from many parts of Washtenaw county (8 zip codes), six zip codes in Wayne county, including several participants from Detroit, and potentially part of Livingston county (one zip code overlaps Washtenaw and Livingston counties), though most of these individuals completed only baseline measures. Follow-up questionnaires come from a much more limited geographical area (the two Ypsilanti zip codes plus one immediately adjacent in Wayne county), and those individuals who completed both a baseline and follow-up questionnaire lived exclusively in the two Ypsilanti zip codes.

### Comparison of Participants at Baseline and Follow-up

Due to the small number of interviews completed and the high degree of missing data as shown in Tables 1 and 2, sex and race were neither significant predictors of being in the follow-up group rather than being in the baseline group nor significant predictors of any of the dependent variables tested (e.g., injection frequency, sharing injection materials, condom usage, etc.). The estimated effect of being female was large, with female respondents having two times the odds of male respondents of being in the follow-up group, but the confidence interval was also substantial (0.80 - 4.86). Measurement of the effect of race was impossible due to the lack of data on follow-up participants.

Using the aggregated groups of baseline and follow-up respondents to calculate unadjusted odds ratios of engaging in injection-related or sexual behavior-related HIV risk behaviors showed two statistically significant findings. First, compared to the baseline group, individuals at follow-up were significantly less likely to report giving another IDU a previously used syringe (OR = 0.38, p = 0.042). Second, follow-up participants were more likely to clean their skin with alcohol either before or after injecting than the baseline comparison group (OR = 3.71, p = 0.01).

Other measures of injection-related risk behavior showed non-significant trends in the direction of risk reduction from baseline to follow-up. NEP users at follow-up were less likely to report sharing syringes (OR = 0.66), sharing equipment other than syringes (OR = 0.70), or reusing syringes (OR = 0.34). Follow-up users were also more likely to report exchanging syringes for another individual (OR = 2.77), though this also failed to reach statistical significance. The odds ratio for using bleach to clean needles and works before reusing them was approximately zero, though there were very low rates of reported bleach use in both the baseline and follow-up groups.

Uptake of other NEP services (e.g., referral into treatment and sexual risk reduction counseling) showed mixed, not statistically significant differences. Positive trends included evidence that participants in the follow-up group were more likely to be willing to go to drug treatment (OR = 1.84) and less likely to report having more than one sexual partner (OR = 0.42). The follow-up group was also less likely to report using a condom (OR = 0.76), though this was not significant.

Self-reported willingness to change injection-related HIV risk behavior (i.e., Stages of Change) was analyzed using a simple regression analysis. On average, respondents reported an increase of 0.24 stages from baseline to follow-up, though this increase was not statistically significant. Using a logistic regression model to calculate the unadjusted odds ratio, follow-up participants were 1.55 times more likely to be in a stage other than pre-contemplation than baseline participants, though this was also not statistically significant.

As shown in Table 1, there were very few participants who completed both a baseline and follow-up questionnaire. Due to this extremely limited sample size, findings from these data should be taken as suggestive of larger trends. Compared to baseline measurements, NEP participants reused their syringes significantly fewer times before getting new ones (p = 0.012). No other comparisons reached statistical significance. There were also modest, but not significant, increases in the number of individuals who reported ever having been tested for HIV, as well as increases in the number of individuals who reported cleaning their skin either before or after injecting. There was also a very small decrease in the number of partners reported and a decrease in reported condom use, though both failed to achieve statistical significance. The data also suggest that participants at both time points are heavy injection drug users averaging more than one injection per day.

## Discussion

The HARC NEP is a relatively small program that fills a critical but often unrecognized need in the community. The HARC Harm Reduction website states that: "Many people think injection drug use (IDU) is not a problem where we live. However, just under 25% of the AIDS cases in HARC's region can be attributed to IDU. It is estimated that between 60% to 80% of all IDU (injecting drug users) are Hepatitis C positive [14]." Though systematic data collection is difficult within small non-profit organizations, analysis of the effectiveness of this type of program is important.

The data presented here suggest that a NEP can be an effective method of harm reduction even in low-volume non-urban settings. While many of the findings were not statistically significant given the small sample size and relatively poor data quality, the consistently positive trends in support of NEP in this setting are suggestive of meaningful behavior change. The results suggest that NEP participation may decrease some injection risk behaviors for HIV including using syringes previously used by another IDU, giving used syringes to other IDUs, and sharing injection equipment other than syringes. That the frequency of injection does not seem to change significantly from baseline to follow-up suggests that individuals using NEP programs are not yet ready to enter treatment and substantially change their drug use behaviors. This finding reinforces the importance of providing harm reduction materials and resources to this population. It is notable that individuals who may enter treatment (and stop using the NEP) are not captured in any of the follow-up measures, so the preliminary results documented here should be interpreted with the understanding that those individuals who have benefited most from this harm reduction intervention are not included in the follow-up sample.

In addition to clean needles, the NEP provides other injection-related harm reduction materials and resources such as cotton, cookers, bleach, and alcohol to clean the skin. In this sample, NEP participation increased the use of alcohol to clean skin when injecting drugs and this increase was statistically significant. Provision of these other resources is an important infection-control mechanism, and is suggestive of a positive effect of NEP participation on injection site infections.

NEP participants were encouraged to reduce sexual risk as well as injection risk of HIV through pre- and post-test counseling and casual interactions with outreach van staff. Only two participants at follow-up reported not having had an HIV test and, although there was missing data from the baseline group (30 individuals did not report whether they had had an HIV test previously), all those who answered the question responded that they had been tested. This finding reinforces previous work suggesting that NEP users are often the highest risk injection drug users, and indicates that the NEP is doing an excellent job promoting HIV testing among those users who had not been tested prior to entering the program.

The observed decrease in condom use between baseline and follow-up is potentially disturbing, as NEPs often emphasize safer sex behaviors as well as safer injection behaviors. The statistically insignificant decrease in condom use may be explained by the decrease in the number of partners; the limited sample size should also temper interpretation of this finding. Nonetheless, this finding may warrant more specific evaluation of sexual risk behavior outcomes of this NEP and indicate that a stronger emphasis is needed on safer sex practices among IDUs, even among those in monogamous relationships, if their needle sharing practices put themselves or their partners at risk.

There are significant limitations to this evaluation. Multiple questionnaire versions make comparisons difficult. Both the matched and aggregate comparisons likely underestimate the program's effect. As was noted earlier, those individuals who entered treatment and stopped using drugs were not captured at follow-up, diminishing the observed effect of the program. A more powerful study with a larger sample size would likely have shown that non-significant differences in injection-related risk behavior in the present study do, in fact, represent substantial behavior change. The aggregate comparisons also likely underestimate the effect of the program because the variables were dichotomized. If the data had been collected consistently with an ordinal scale, it would have been possible to use the full range of data reported. Instead, information was lost when the categories were collapsed into "never" and "ever," though this was necessary since not all respondents answered using the same scales. In addition, the small sample size is likely a result of not only observing a particularly transient population, but also a focus on the part of HARC staff on the provision of services and not necessarily evaluation. Given their limited time and resources, this allocation makes sense. In spite of these limitations, these data provide important and unique evaluation outcomes from a peri-urban NEP. For this reason, these findings are an important contribution to the literature evaluating the effectiveness of needle exchange programs.

## Conclusions

Overall and despite the limitations, this evaluation shows the promise of small-scale needle exchange programs in areas not served by large urban needle exchange programs. Even using very small samples and dichotomized measures, this evaluation showed that a small peri-urban needle exchange program can contribute to reducing injection-related HIV risk behavior.

## Competing interests

The authors declare that they have no competing interests.

## Authors' contributions

AK participated in the design of the study, entered the data, performed the statistical analysis, and drafted the manuscript. PW participated in the design of the study and helped to draft the manuscript. LG participated in the gathering of the data, interpretation of the results, and revision of the manuscript. All authors read and approved the final manuscript.
